# Duodenum Exclusion Alone Is Sufficient to Reduce Fasting Blood Glucose in Non-Obese Diabetic Goto-Kakizaki Rats

**DOI:** 10.1007/s11695-013-1167-9

**Published:** 2014-01-05

**Authors:** Jun Ke, Yu Wang

**Affiliations:** 1 Department of General Surgery, Fuzhou General Hospital, Fujian University of Traditional Chinese Medicine, Fuzhou, Fujian 350025 China; 2 Department of General Surgery, Fuzhou General Hospital of Nanjing Command, Fuzhou, Fujian 350025 China

To the editor,

In addition to reducing weight, bariatric surgery could improve glucose metabolism in patients with type 2 diabetes mellitus [1, 2]. Such actions have mainly been explained by the foregut and the hindgut hypotheses. The foregut hypothesis claims that bariatric surgery removes endogenous substances that cause disturbance in glucose metabolism [3]. The hindgut hypothesis postulates that the glucose-lowering action is mainly the result of the expedited food delivery to the hindgut [4]. However, effects of gastric bypass surgery on glucose metabolism and the relative contribution of the foregut vs. hindgut hypothesis vary considerably based on the type of the surgery. Another major confounding factor is the reflux of gastrointestinal (GI) content into the duodenum [5, 6].

**Fig. 1:**
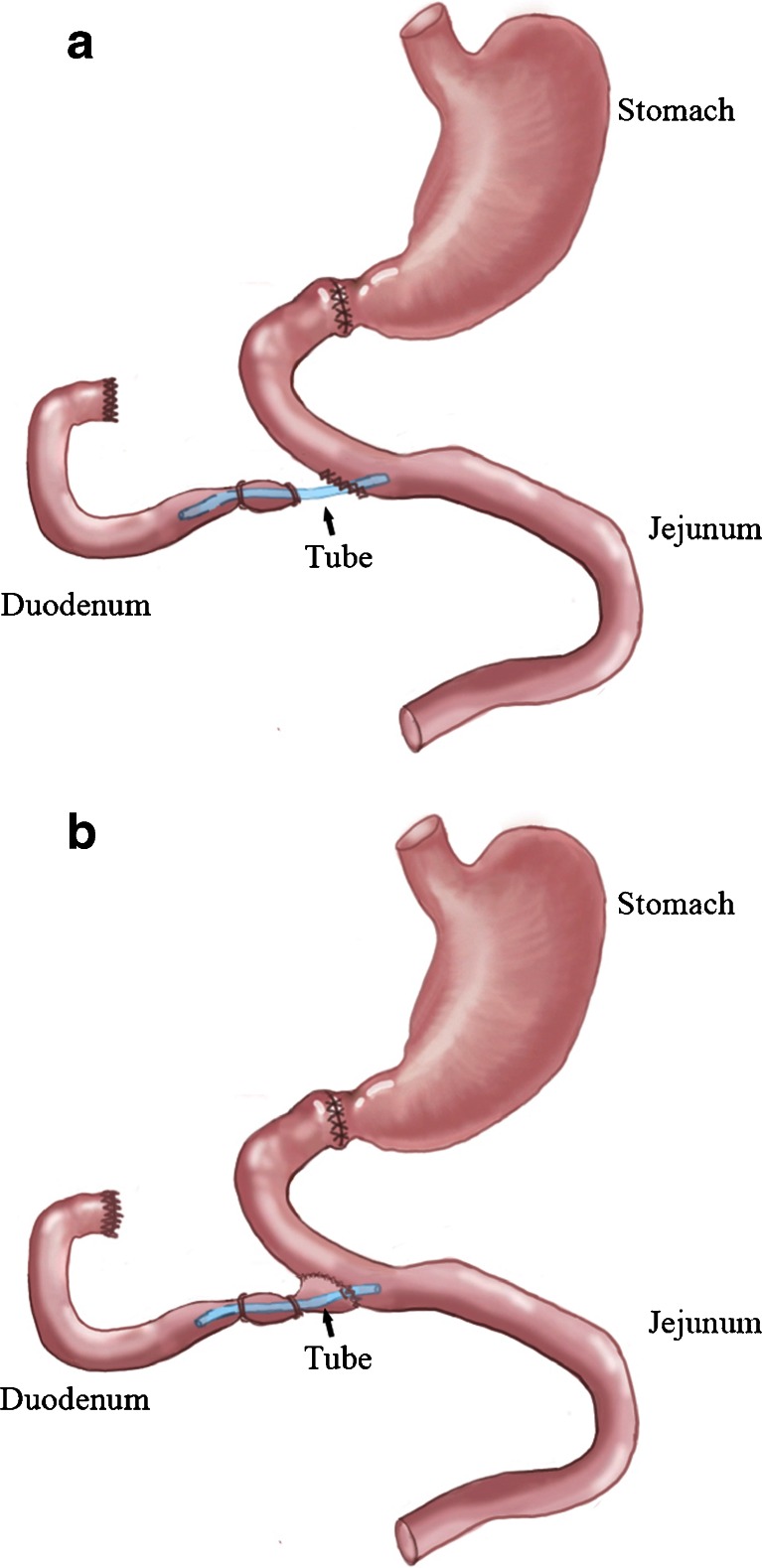
A schematic illustration of duodenum exclusion (**a**) and modified duodenum exclusion (**b**)

**Fig. 2:**
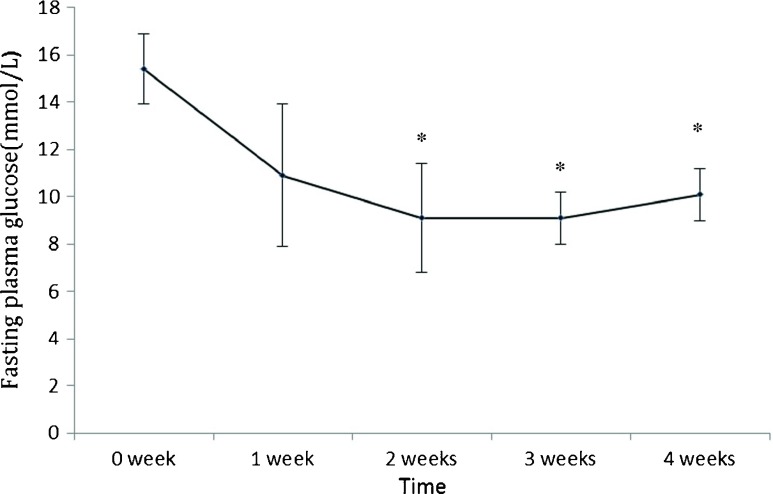
Data are expressed as mean ± SD and analyzed with pairwise *t* test. *p < 0.05 vs. presurgery baseline

In this preliminary rat study, we examined whether duodenum exclusion alone is sufficient to improve glucose metabolism in a rat model of diabetes. Briefly, the intestine of non-obese diabetic Goto-Kakizaki (GK) rats was transected immediately below the ligament of Treitz. A 3-mm silicon tube was used to connect the duodenum with jejunum and fixed in the wall of jejunum (Fig. 1a). Short-term mortality (<1 week) in this pilot experiment was 100 % (*n* = 55; Table 1). We therefore modified the procedure by wrapping a shorter silicon tube in the duodenum and ligation of the distal end of the duodenum (*n* = 20; Fig. 1b) and achieved a 90 % survival (18/20). Such a modified surgery resulted in a significant reduction of fasting plasma glucose in the following weeks (Fig. 2).

**Table 1:** Cause of death

Type	Duodenum exclusion	Modified duodenum exclusion
Anesthesia	1	0
Bleeding	4	0
Fistula	5	0
Adhesion	41	0
Intestinal obstruction	1	2
Unknown	3	0
Total	55	2

In this study, the surgical intervention completely excluded the duodenum and prevented the possibility of reflux. The results showed that duodenum exclusion alone is sufficient to reduce blood glucose. To further examine the validity of the model, we are currently conducting a series of experiments to expand the finding to other relevant measures (such as insulin tolerance) and to examine the effects of establishing the normal GI integrity in rats with duodenum exclusion.

In summary, this surgery is challenging but feasible for experienced surgeons and may provide an alternative animal model to study the action of bariatric surgery.
